# The Association of Medical Officers of Hospitals for the Insane

**Published:** 1851-10-01

**Authors:** 


### THE ASSOCIATION OF MEDICAL OFFICERS OF HOSPITALS FOR THE INSANE.

We subjoin the Official Report of the meeting of the Association, held in London on the 17th of July last. At this meeting, the Association was partially reorganized. According to the rules of the society, all medical superintendents of public asylums were *ex officio* members of the Association, and its meetings were confined to the provinces. Agreeably to the new arrangements, medical gentlemen connected with *private* asylums have been admitted into fellowship with the Association, and there are to be quarterly meetings of the society in London, as well as an annual meeting in the month of July, and a metropolitan as well as a provincial secretary has been appointed.

We congratulate our readers on this liberal and necessary enlargement of the boundaries of the society. We sincerely trust that no event will occur to interfere with the usefulness of this Association. It has great objects to accomplish. It should, in every sense of the word, be a *working body*, watching with proper jealousy the progress of public opinion in reference to the insane, and thwarting, to the best of its ability, the introduction of legislative measures calculated to cripple the hands of those entrusted with their care, thus retarding the advancement of mental pathology, and inflicting a serious injury upon the cause of humanity.

If the influential body of able and intelligent men connected with this Association act in concert, and are influenced by the same enlarged and humane views, it will be impossible to estimate the good that may result from their combined operations. We have important objects to effect, principles to establish, and common interests to protect. It is therefore the sacred duty of each individual member of the Association to put his shoulders to the wheel, and do his utmost to co-operate with his brother members in the furtherance of a cause so closely connected with the advancement of an important branch of medical science, and the well-being of the human race.

No private feelings, no mistaken views of the course to be pursued, no professional jealousy, should for one moment interfere with that *unanimity of action* so essential to the successful working, if not to the very existence, of the Association. We have enemies *outside* of the camp ready to take immediate advantage of any disunion in our own ranks; let us not be guilty of the folly of extinguishing ourselves by an act of suicide, by engendering and encouraging internal feuds and dissensions. As an Association, our actions should place us beyond all cavil and suspicion. Let us beware of the fatal rock against which so many kindred societies have, alas! been lamentably wrecked. As a first principle, the repudiation of all attempts to form that most odious and destructive of bodies, *a clique*, should steadily, resolutely, and perseveringly be carried out. As an Association, we meet as *equals*. Recognising the respect due to talent, knowledge, and character, we must indignantly refuse to bow the knee to mere *names*. If we commit the fatal mistake of permitting any one member, or even a small section of our body, to exercise undue influence or control over its deliberations, we reduce ourselves to a contemptible position, and surrender, what ought to be dear to every Englishmen—the right of thinking for ourselves. Should such be the unhappy result, then for all useful purposes the Association has ceased to exist! High and noble impulses ought alone to actuate, guide, and urge us on. We must have no private ends to serve—caprices to gratify—wrongs to redress. If differences of opinion arise—and differences we must expect—let them be openly expressed, and then calmly and dispassionately considered; but beware of allowing the important, the vast interests at stake to be perilled by the injudicious—we were on the eve of saying, imbecile—submission to the authority of the few ever ready to over-ride and lead by the nose those unfortunately weak enough to blindly prostrate themselves at the shrine of any idol that may have obtained a factitious and temporary elevation. *The Association of Medical Officers of Hospitals for the Insane* must not consist of *toadies* and *tuft-hunters*, men possessing the *minimum* amount of intellect and knowledge, conjoined with the *maximum* degree of ignorance, arrogance, and presumption;—but of intelligent, educated members of a liberal and enlightened profession, engaged in the solemn prosecution of a just and holy undertaking.

With these suggestions, we proceed to lay before our readers the Official Report of the secretaries.

At a meeting of the Association of Medical Officers of Hospitals for the Insane, held at the Freemasons' Tavern, in London, on Thursday, July 17th, 1851, the following members were present;—Dr. John Conolly, visiting physician, County Middlesex Hospital for the Insane at Hanwell; Dr. Richard Lloyd Williams, do., North Wales Hospital for the Insane, Denbigh; Dr. Kirkman, resident physician, County Suffolk Hospital for the Insane; Dr. Nesbitt, do., Northampton do.; Dr. Bucknill, do.; Devon do.; Dr. Begley, resident surgeon, County Middlesex do.; Mr. Diamond, do., County Surrey do.; Dr. H. Ramsay, Gloucester do., Dr. Stewart, resident physician, District Hospital for the Insane, Belfast; Dr. Boyd, do., County Somerset do.; Mr. Prosser, resident medical superintendent, Leicester do.; Mr. Alderson do., Nottingham do.; Dr. Stewart Allen, resident physician of the Marylebone Hospital for the Insane; Mr. Ley, resident medical superintendent, Oxford and Berks do.; Dr. Wintle, do., Warneford Hospital for the Insane, Oxford; Mr. Eccleston, do., West Derby County Hospital for the Insane, Rainhill, Prescot; Dr. Forbes Winslow, Hammersmith; Dr. Henry Monro, Mayfair; Dr. William Conolly, Hayes Park; Mr. Cornwall, Fairford, Gloucester; Mr. Mallam, Hooknorton, Oxon; Dr. Bush, Sandywell Park, Cheltenham; Mr. Ogilvie, Bristol; Dr. Bascombe, Wyke House, Brentford; Dr. Cox, Fishponds, Bristol.

Dr. JOHN CONOLLY, being the senior member present of the Metropolitan Hospitals for the Insane, was requested to take the chair.

Dr. STEWART, Secretary of the Association for Ireland, was requested to act as secretary to the meeting. The minutes of the last meetings, held in Oxford, were read and confirmed, and signed by the chairman. Letters were read from Drs. Hitch and Williams, of Gloucester, the secretaries, and Dr. Oliver, of the Salop Hospital for the Insane, regretting their inability to attend the meeting. Dr. Hitch having in his letter expressed a wish to resign the office of joint-secretary, his resignation was accepted, a vote of thanks being unanimously voted to him for his long-continued and valuable services, and, at the same time, it was resolved that he should be requested to continue his services as the treasurer of the Association.

The usual Report made by the secretaries was read and adopted.

The Chairman briefly and ably addressed the meeting concerning the objects of the Association, and pointed out some of the services it might usefully perform, such as the revision of the Lunacy Acts, &c.

Dr. Kirkman, with much effect, illustrated the necessity of a revision of the Acts.

Dr. Lloyd Williams supported Dr. Kirkman's judicious and apposite observations, and mentioned instances in which the dissent of counties united in one asylum was not provided against, and obstructed improvements.

Mr. Ley, Mr. Alderson, and Dr. W. Conolly, respectively directed attention to the necessity there existed for an improved state of the Acts of Parliament relating to lunacy.

It was then resolved unanimously,—"That a committee be formed to examine the Lunacy Acts, and to draw up a report thereon, indicating ambiguities and defects, and suggesting alterations and amendments; such report to be printed and circulated among the members of the Association for their adoption, and subsequently transmitted to the Secretary of State and to the Commissioners in Lunacy; and that the above committee consist of the following members:—Dr. J. Conolly, Dr. Forbes Winslow, Dr. Bucknill, Dr. Hitch, Dr. Nesbitt, Dr. Boyd, Dr. Corsellis, and Dr. Diamond."

Dr. Forbes Winslow was requested to act as secretary to the committee, and to apply for and arrange into proper form such written statements as might be supplied on the subject for which it was appointed, which he consented to do, it being understood that although the above committee undertook to apply themselves especially to the object for which it was formed, yet the assistance and suggestions of all the members of the Association would be willingly received.

The Chairman read an extract from a letter addressed to him by Dr. Williams, of the Gloucester Hospital for the Insane, urging the propriety of a petition being addressed by the Association to Parliament, for the establishment of a Central Criminal Asylum, and observing that such an asylum had been established by Act of Parliament in Ireland, and found to work admirably. The Chairman, with much feeling and ability, alluded to the unfavourable position in which criminals were now placed if the plea of insanity was admitted, their doom being actually worse than transportation, and almost worse than death.

Dr. Begley said he had visited the Central Lunatic Asylum, at Dundrum, near Dublin, and found it excellently adapted to its purpose.

Dr. Stewart stated that he also had been visiting the Central Asylum, which he considered admirably circumstanced in all essential respects for such an institution, its situation, too, being most cheerful and picturesque, and its whole management most ably and humanely conducted by Dr. Corbett, the resident physician, appointed by government. After observing upon the great relief now afforded to the hospitals for the insane in Ireland, by the criminal insane being placed in an entirely distinct establishment, he presented to the Association, in the name of the Government Inspectors of Asylums in Ireland—Drs. White and Nugent—their "Fifth General Report on the District Criminal and Private Lunatic Asylums in Ireland," containing full particulars and statistics of the Central Asylum, in common with all the other asylums in that country.

Dr. FORBES WINSLOW supported the necessity for a central asylum in England, where the criminal lunatics might be properly classified, concluding his observations on this subject by alluding with much energy and feeling to the indignities and browbeatings which of late especially, medical witnesses of the greatest eminence and respectability were subjected to, both from the bench and by the bar, when called upon to give evidence in support of the plea of insanity in criminal cases.

Dr. KIRKMAN advocated the importance of a criminal asylum being established, and strongly advised that the Association should lose no time in following the example of Ireland in a matter of so much importance, and so much affecting the comfort and well-being of the ordinary inmates of hospitals for the insane.

Drs. NESBITT and BUCKNILL, having expressed similar views, and shown the inconveniences and injustice of present arrangements for criminal lunatics, it was unanimously resolved,—1. That it is desirable that there should be a Central Asylum for Criminal Lunatics in England, distinct from any asylum in which the insane, not criminal, are received. 2. That Dr. Williams, of the Gloucester Hospital for the Insane, be requested to give his best consideration to this subject, and to prepare a petition, to be submitted to the members of this Association, and to be forwarded to the Secretary of State for the Home Department. Resolved unanimously, that the especial thanks of the Association be given to Drs. White and Nugent, the Government Inspectors of Hospitals for the Insane in Ireland, for their courtesy in now presenting the Association with their lately published valuable annual Report, and for the great improvements they have been so instrumental in effecting in the District Hospitals for the Insane in Ireland, by obtaining the decision of Government in favour of none but medical men, duly qualified and experienced, being for the future appointed the resident superintendents of those important public institutions. Also, resolved unanimously, that Drs. White and Nugent be elected members of this Association, and that a copy of the foregoing resolution be duly transmitted to them by the secretaries.

The following resolutions were also agreed to:—That the Annual meeting of the Association be in future held in London, on the second Saturday of July, in each year, at the Freemasons' Tavern, at one o'clock, P.M. That quarterly meetings, of such members of the Association as can conveniently attend them, be held on the first Saturday in the months of March, June, September,* and December, in each year, at three o'clock, P.M. (Dr. Forbes Winslow offered the use of his rooms, in Albemarle-street, for those meetings.) That Dr. Diamond, of the Surrey Hospital for the Insane, be requested to act as Metropolitan Secretary. That Dr. Williams, of the Gloucester Hospital for the Insane, be requested to continue his valuable services as Secretary. That the annual subscription for the purpose of defraying unavoidable expenses, be five shillings.

Mr. ALDERSON, of the Nottingham Hospital for the Insane, exhibited and explained an improved lock-button, for the purpose of securing the dress of the insane, for which the thanks of the meeting were voted to him.

It was proposed, and agreed to, that the Association visit the new Additional Hospital for the Insane for the county of Middlesex, at Colney Hatch, on next day (Friday), at one o'clock, P.M.; also, the Asylum for Idiots, at Highgate, on Saturday forenoon, the 19th instant; and, on the invitation of Dr. Diamond, the Surrey Hospital for the Insane, in the

* We are now writing on the 24th of September, and no quarterly meeting has been held—at least, the Editor of this Journal has received no summons to attend one. Who is responsible for this neglect?

afternoon of the same day; also to visit, on the same day, Bethlem Hospital.

All the gentlemen present were voted members of the Association, as also, Dr. Stillwell, of Hillingdon; Dr. Tuke, Chiswick; Mr. Denne, Hanwell; Dr. Daniel, Southall Park; Dr. White, resident physician, Carlow District Hospital for the Insane; Dr. Corbet, ditto, Central Asylum, Dundrum; Dr. Power, visiting physician, Cork District Hospital for the Insane; Dr. Hood, resident physician, Colney Hatch; Mr. Snape, resident surgeon, Surrey Hospital for the Insane; Mr. Green, resident medical superintendent, Borough Hospital for the Insane, Birmingham; Dr. Pritchard, Abington Abbey, Northampton; Dr. Maxwell, resident physician, Asylum for Idiots; Dr. Wood, resident physician, Bethlem Hospital.

It was resolved unanimously—that the cordial thanks of the meeting be given to Dr. Stewart, of the Belfast Hospital for the Insane, the Secretary of the Association for Ireland, for his attendance and valuable services at the meeting.

Dr. Conolly having left the chair, and Dr. Lloyd Williams having taken it, it was resolved unanimously—That the marked thanks of this meeting be given to Dr. Conolly for the very able and courteous manner in which he has fulfilled the duties as Chairman of this meeting.

*First Adjourned Meeting at Colney Hatch New Hospital for the Insane for the County of Middlesex, on Friday, July 18th.*

Present.—Dr. Hood, Dr. Davey, resident physicians of the hospital; Mr. Mallam, Dr. Kirkman, Dr. Wintle, Dr. Ramsey, Dr. Boyd, Mr. Eccleston, Mr. Alderson, Mr. Ogilvie, Dr. Richard Lloyd Williams, Mr. Ley, Mr. Cornwall, Dr. Bush, Dr. Stewart. Visitors.—Mr. Nunneley, F.R.C.S., Leeds; Mr. Moseley, architect of the New Hospital for the Insane for the County of Lancaster, at Rainhill, Prescot, and the Surrey ditto, and the New Wings at Hanwell.

The members being conducted through the entire establishment by Doctors Hood and Davey, both gentlemen were thanked for their attentions on the occasion, and Dr. Hood for the hospitalities he had provided for the Association.

A conversation having arisen amongst the members of the Association, after the above meeting, respecting the address which had been presented by them, some time since, to Mr. Gaskell, late medical superintendent of the County Asylum at Lancaster, congratulating him on his appointment as one of the Commissioners in Lunacy, and to which no answer had been supposed to have been received to the present time, but which it now appeared had been duly given, it was resolved,—That Dr. Williams, Gloucester, be respectfully requested to have the address above referred to, inserted, with Mr. Gaskell's reply, in the proceedings of the Association, and printed at the same time with the minutes of this year's meetings, for circulation amongst the members.

*Second Adjourned Meeting at the Asylum for Idiots, Park House, Highgate, on Saturday, July 19th.*

Present.—Dr. Maxwell, resident physician of the asylum; Dr. Ramsey, Mr. Eccleston, Dr. Bucknill, Dr. Wood, resident physician, Bethlem Hospital; Dr. Stewart.

Dr. Maxwell having kindly brought the Association through the respective divisions, and over the grounds of the institution, they took their leave, much pleased with Dr. M.'s attention whilst conducting them over the establishment.

The Association were received last evening by Dr. Forbes Winslow, at a *conversazione,* at his residence, Sussex House, Hammersmith, a large number of members and others being present on the occasion.

*Third Adjourned Meeting at the Surrey County Hospital for the Insane, Wandsworth, on Saturday, July* 19*th.*

Present.—Dr. Diamond, Mr. Snape, resident medical officers; Dr. Lloyd Williams, Mr. Eccleston, Mr. Ley, Mr. Alderson, Dr. Ramsey, Dr. Wood, Dr. Stewart.

Dr. Diamond and Mr. Snape conducted the members in attendance through this large establishment, when a vote of thanks was passed to them for their attention, &c.; after which the Association withdrew.

*Fourth Adjourned Meeting at Bethlem Hospital, on Saturday, July* 19*th.*

Present.—Dr. Monro, visiting physician, and Dr. Wood, resident physician of the hospital; Dr. Richard Lloyd Williams, Dr. Ley, Mr. Eccleston, Dr. Bucknill, Dr. Ramsey, Dr. Kirkman, Dr. Stewart.

The members visited the several departments of the establishment, and were shown a neat pattern window-frame, fitted up in one of the corridors, and also a bed in use in the hospital for wet patients, both being the designs of Dr. Wood, and both appearing well calculated for their respective purposes.

The members of the Association thanked the medical officers for their attention on the occasion of their present visit; and being honoured with, and having accepted of, an invitation to dinner by Dr. Wood, finally took their leave, much pleased with all the attentions they had received.

---

